# Measuring the atomic spin-flip scattering rate by x-ray emission spectroscopy

**DOI:** 10.1038/s41598-019-45242-8

**Published:** 2019-06-20

**Authors:** Régis Decker, Artur Born, Robby Büchner, Kari Ruotsalainen, Christian Stråhlman, Stefan Neppl, Robert Haverkamp, Annette Pietzsch, Alexander Föhlisch

**Affiliations:** 1 Institute for Methods and Instrumentation for Synchrotron Radiation Research FG-ISRR, Helmholtz-Zentrum Berlin für Materialien und Energie Albert-Einstein-Strasse 15, 12489 Berlin, Germany; 2 Institut für Physik und Astronomie, Universität Potsdam, Karl-Liebknecht-Strasse 24-25, 14476 Potsdam, Germany

**Keywords:** Magnetic properties and materials, Electronic properties and materials, Ferromagnetism

## Abstract

While extensive work has been dedicated to the measurement of the demagnetization time following an ultra-short laser pulse, experimental studies of its underlying microscopic mechanisms are still scarce. In transition metal ferromagnets, one of the main mechanism is the spin-flip of conduction electrons driven by electron-phonon scattering. Here, we present an original experimental method to monitor the electron-phonon mediated spin-flip scattering rate in nickel through the stringent atomic symmetry selection rules of x-ray emission spectroscopy. Increasing the phonon population leads to a waning of the 3*d* → 2*p*_3/2_ decay peak intensity, which reflects an increase of the angular momentum transfer scattering rate attributed to spin-flip. We find a spin relaxation time scale in the order of 50 fs in the 3*d*-band of nickel at room temperature, while consistantly, no such peak evolution is observed for the diamagnetic counterexample copper, using the same method.

## Introduction

The experimental determination of the *microscopic* spin-flip scattering rate in solids is of fundamental importance in order to better understand their *macroscopic* properties such as the femtosecond demagnetization process^1^. However, while more than two decades of experimental work have been devoted to quantify the ultrafast demagnetization time constants using mainly pump-probe strategies, the experimental quantification of the spin-flip rates appears to be more challenging and therefore, scarcely investigated. More specifically, the femtosecond demagnetization of ferromagnets^2^, the transient states in ferrimagnets^3^ or the modifications of antiferromagnetic order^4^ have common microscopic physical drivers. Among these drivers are the atomic electron-phonon^5–10^ and electron-magnon^11^ mediated spin-flip scattering, non-collinear momenta reordening^4^, different velocities of minority and majority spin electrons in superdiffusive spin transport^2,12^ or intersite spin-selective charge transfer^13^. All these drivers satisfy the boundary condition of angular momentum conservation within accessible spin, electron orbital and lattice degrees of freedom of a material.

Here, we present a method to determine the temperature-dependent atomic electron-phonon induced spin-flip scattering rate. We exploit the quantifiable change in the decay peak intensities in static x-ray emission spectroscopy (XES) spectra when changing the temperature, *i*.*e*. when changing the phonon population. We apply this method to nickel and copper as test model systems. For nickel we observe a decrease of the intensity of the emission peak corresponding to the spin-polarized 3*d* valence band to the created 2*p*_3/2_ core hole. We interpret this decrease as a result of the Elliott-Yafet type spin-flip scattering of valence electrons with phonons, which reduces the decay probability. Accordingly to our interpretation, the diamagnetic counterexample copper presents no temperature dependance of the decay peak.

**Figure 1:**
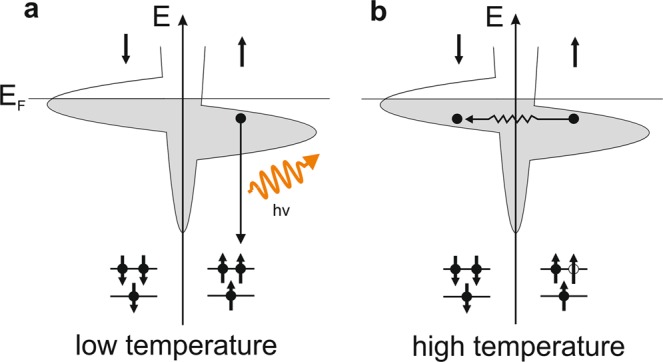
Schematic principle of the XES process in the presence or absence of spin-flip scattering in nickel after the creation of a core-hole. (**a**) Low temperature case: radiative decay from the filling of a core hole by a valence band electron. (**b**) High temperature case: spin-flip processes induced by electron-phonon scattering events reduce the radiative decay probability of the core-hole.

The basic underlying idea of the method is illustrated in the simplified schematics of Fig. 1, which depicts the radiative decay from a valence band electron to a created core-hole. At low temperature this decay occurs during the core-hole lifetime from electrons having the same spin. At high temperature, electron-phonon scattering-driven angular momentum transfer events can flip the electron spin and lead to a lower radiative decay rate, visible as a lower corresponding peak intensity in XES spectra.

## Results and Discussion

### Temperature-dependent x-ray emission spectroscopy

**Figure 2:**
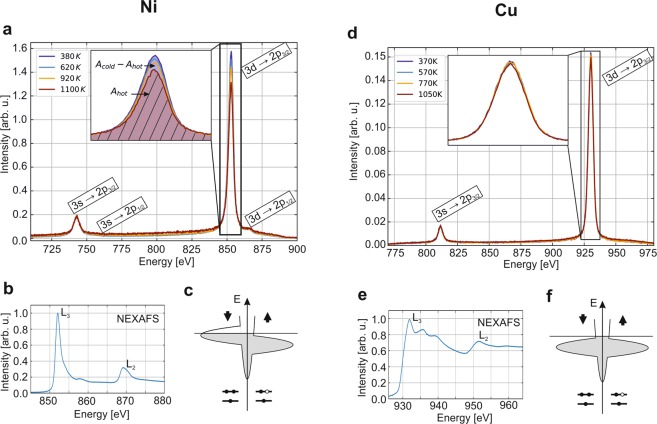
Temperature dependence of the XES spectra of nickel and copper. (**a**) Temperature-dependent XES spectra of nickel. An increase in the temperature leads to a decrease in the 3*d* → 2*p*_3/2_ peak intensity. (**b**) The room temperature NEXAFS spectrum of nickel recorded during the experiments shows no indication of an oxide. (**c**) Schematically illustrated nickel valence density of states (DOS) with the 2*p* core levels. The magnetic properties arise from the half-filled spin minority 3*d* band. (**d**) XES spectra of copper. Here, the 3*d* → 2*p*_3/2_ peak intensity is temperature-independent. (**e**) The room temperature NEXAFS spectrum of copper recorded during the experiments shows no indication of an oxide. (**f**) Schematically illustrated copper valence DOS with the 2*p* core levels. Due to the fully occupied bands, copper is diamagnetic.

We apply this method to the 3*d*-ferromagnetic model system nickel. The experimental data is presented in Fig. 2(a), which shows XES spectra taken with an incident energy of *h*ν = 865 eV. At this chosen incident photon energy, selectively a Ni 2*p*_3/2_ core level vacancy is created, which decays within the natural Ni 2*p*_3/2_ core level life time of *τ*_*core*−*hole*_ = 1.04 fs^14^. The incident energy is chosen well above the Ni L_3_ edge in order to excite the core electron to the continuum and to be in the non-resonant regime. This allows probing the weakly perturbed valence band when measuring the radiative decay. The radiative decay of the Ni 2*p*_3/2_ core vacancy through valence electrons within XES obeys the atomic dipole selection rules of Δ*l* = ±1 and Δ*s* = 0. Thus, we detect within the 700 eV to 900 eV photon emission energy range of our X-ray spectrometer simultaneously the 3*s* → 2*p*_3/2_, and the 3*d* → 2*p*_3/2_ transitions. Note that the 3*s* → 2*p*_1/2_, and the 3*d* → 2*p*_1/2_ transitions are also slightly visible. A 3*p* → 2*p* non-dipole x-ray emission arising due to resonant Raman scattering was reported previously^15^. However, its spectral signature is not visible in our data.

Raising the temperature of the nickel crystal leads to a waning of spectral intensity of the Ni 3*d* → 2*p*_3/2_ transition, as highlighted in the inset of Fig. 2(a). The partially occupied Ni 3*d* valence states can undergo changes in orbital and spin character, due to low energy scattering events with phonons. Thus, the initially atomically prepared state of the Ni 2*p*_3/2_ core vacancy that is radiatively filled by the sub-set of dipole allowed Ni 3*d* electrons, is sensitive to Ni 3*d* electron-phonon scattering, which in particular changes the spin state of the valence electrons. However, for the Ni 3*s* inner valence state, that is fully occupied, no change of angular momentum and spin state can occur and a constant spectral intensity *vs*. the temperature for the radiative transition into the Ni 2*p*_3/2_ is expected. Therefore, the peak area of this transition is assumed to be constant and is used to normalize spectra. More precisely, the entire spectra are multipied by a factor in order to keep this peak area, after subtracting the background area under it, constant for all temperatures^16^. The background comes mainly from the glowing filament and the warm parts around the samples, when measuring at high temperature during several hours. What is shown in Fig. 2 is the normalized spectra including the total background. Figure 2(b) shows a nickel Near-Edge X-ray Absorption Spectroscopy (NEXAFS) spectrum measured in the total electron yield mode and acquired during our experiments. The energy range includes the *L*_2_ and *L*_3_ edges at 871.9 eV and 854.7 eV, respectively^17,18^. Our NEXAFS data correspond to those expected for clean nickel^19^. In particular, the satellite peak at 859 eV, known as the 6-eV feature, which arises from strong electronic correlation effects, is visible^20^.

To elucidate the aspect of spin-flip scattering further, we performed similar experiments on copper, where we can create the analogous Cu 2*p*_3/2_ core-vacancy. In contrast to nickel, the Cu 3*d*-band as well as the Cu 3*s* inner valence state are fully occupied. Thus, no spin-flip scattering is possible. Figure 2(d) shows the temperature-dependent XES spectra of copper for an incident energy of 945 eV, *i*.*e*. between the *L*_2_ and *L*_3_ edges. Here, both the radiative decay of the fully occupied Cu 3*s* inner valence and the Cu 3*d*-band into the atomic Cu 2*p*_3/2_ core-vacancy leads to no detectable changes in spectral intensities with the temperature. Since the Cu 2*p*_3/2_ core hole life time is with *τ*_*core*−*hole*_ = 0.56 fs^14^ rather similar to the one of nickel, this cannot be attributed to a shorter scattering duration time for copper than for nickel. And again, the NEXAFS spectrum of copper (Fig. 2(e)), which presents the *L*_2,3_ peaks at 952.3 eV and 932.7 eV in addition to two distinct satellite peaks at 937.6 eV and 941.5 eV, is characteristic of clean copper with no indication of the presence of oxide or other contaminants^21^.

## Discussion

The evolution of the 3*d* → 2*p*_3/2_ peak with the temperature is the consequence of a reduction of the density of 3*d* electrons available for the decay to the created core-holes. This reduction can be the result of either electrons with a 3*d* symmetry being excited to a 4*s* or 4*p* symmetry or of a spin-flip of the 3*d* electrons. Both scenarios can originate from an electron-phonon angular transfer. The first scenario is unlikely since (i) 4*s* and 4*p* density of states (DOS) are more than an order of magnitude smaller than the 3*d* DOS and would not explain a visible change in the XES peak intensity and (ii) it is not consistent with an absence of temperature dependence of the XES spectra for copper, where such excitations could also be considered. Therefore, the XES peak evolution is more likely the result of the spin-flip of 3*d*-electrons, which is allowed for nickel but not for copper. Spin-flip transitions of localized 3*d* electrons in nickel driven by spin-orbit coupling have been recently proposed as a microscopic mechanism of the demagnetization dynamics^22^.

**Figure 3:**
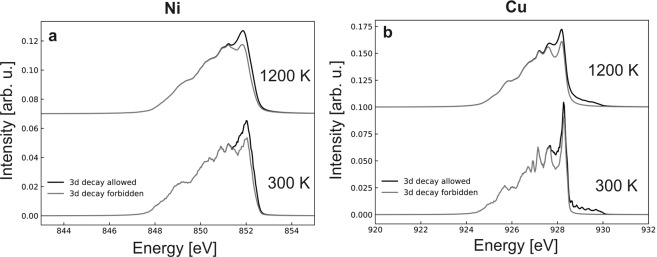
Simulated 3*d* → 2*p*_3/2_ emission peak of (**a**) nickel and (**b**) copper at 300 K and 1200 K. The plots show the change in the emission peak due to the presence or absence of electron decay from the 3*d*-bands crossing the Fermi surface, leaving electrons from all other bands free to decay. This change is shown for 300 K and 1200 K. For clarity, the center of the peak is set to 852 eV for nickel and 928 eV for copper and the plots are shifted vertically for different temperatures.

To test this interpretation, we performed Density Functional Theory (DFT) calculations to simulate the decay peak intensity. Our calculations show a reduction of the peak area of 5.5 % and 5.4 % for nickel and copper, respectively, due to the temperature-induced lattice expansion and the Fermi-Dirac smearing (see^16^). The facts that the value for nickel is smaller than the experimentally observed waning and that we observe very similar values for nickel and copper indicate that the lattice expansion and the Fermi-Dirac smearing only, i.e. without scattering, are not the main contributions to our observations. Simulated 3*d* → 2*p*_3/2_ emission peaks are presented in Fig. 3. The plots show the difference in the emission peak when allowing or prohibiting the decay from the 3*d* bands crossing the Fermi surface. This would be the consequence of an electron scattering within the 3*d* band, reducing the decay probability. We find a reduction of the peak area for both nickel and copper and for low (300 K) and high (1200 K) temperatures. This reduction is in the order of 12 % for nickel and 16 % for copper. This matches only the observed peak reduction of 11 % for nickel at high temperature, consistently with our interpretation. Indeed, the fact that this reduction is not experimentally observed at low temperature indicates electron-phonon scattering. In addition, since the calculated peak reduction for copper is not observed experimentally, this speaks consistently against the possibility of a spin-flip scattering process in copper. Copper has a full 3d band, which prevents spin-flip scattering events, in contrast to nickel, which has a partially filled 3d band, below and above the Curie temperature, and for which spin-flip scattering is allowed.

### Determination of the scattering rate

**Figure 4:**
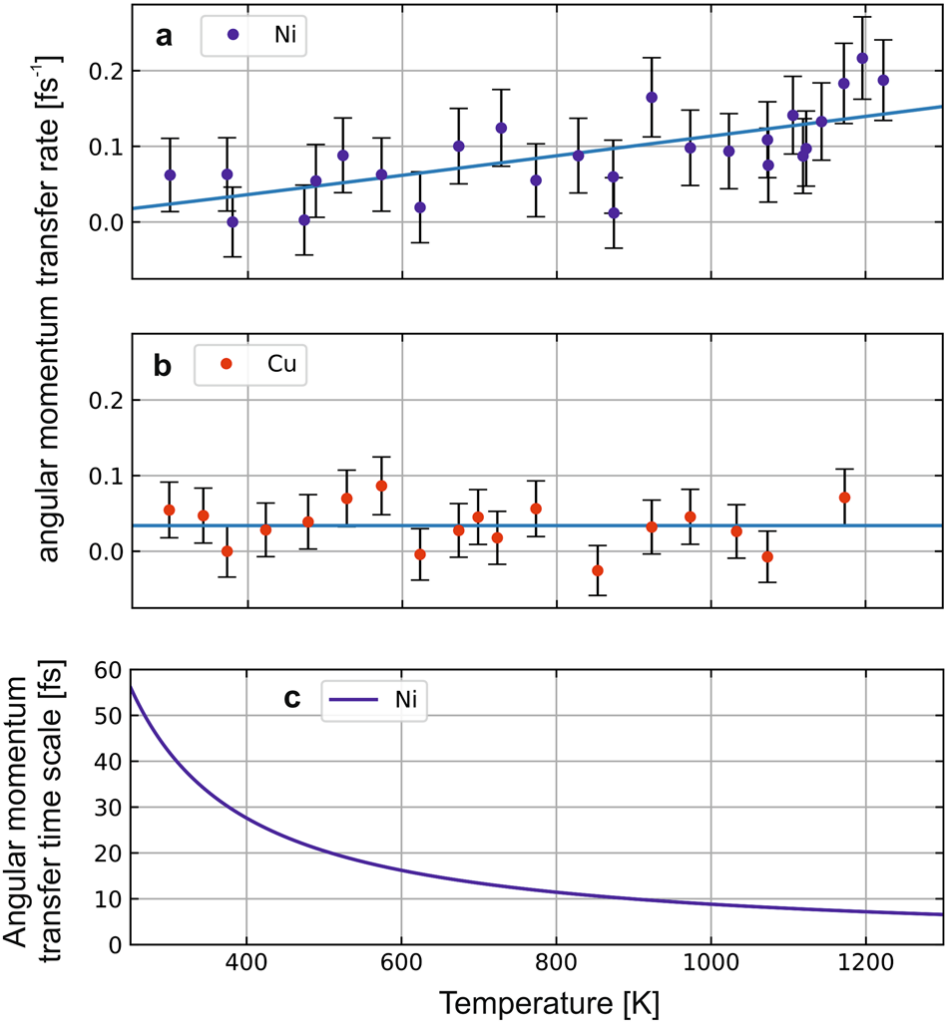
Angular momentum transfer rate. (**a**) nickel. (**b**) copper. Points are experimental data obtained from the XES spectra. Lines are fits. (**c**) Momentum transfer lifetime deduced from the fitted rates in (**a**).

Following our interpretation, we quantify our experimental findings in Fig. 4, where the angular momentum transfer rate of nickel (a) and copper (b) as a function of the temperature are shown in direct comparison. An important remark must be made here about the analysis of the peak area, which consists in a normalization against the 3*s* → 2*p*_3/2_ peak area, as discussed above and which gives the spectra shown in Fig. 2, in addition to a background subtraction. Indeed, after normalization and especially for nickel, we still observe a slight difference in the background signal (see Fig. 2). For the data shown in Fig. 4, in addition to the normalization, we estimated carefully and subtracted this background area below the 3*s* → 2*p*_3/2_ and the one below the 3*d* → 2*p*_3/2_ peaks. Further details about the background subtraction are given in the Supplementary Material^16^.

We established previously in semiconductors how the angular momentum transfer scattering rate *R*(*T*) can be deduced from the evolution of valence to core-hole decay peaks with temperature^23,24^ as:

1 $$R(T)=\frac{1}{{\tau }_{core-hole}}\cdot \frac{{A}_{inc}}{{A}_{coh}}=\frac{1}{{\tau }_{core-hole}}\cdot \frac{{A}_{cold}-{A}_{hot}}{{A}_{cold}}$$

where *τ*_*core*−*hole*_ is the core-hole lifetime of the excited state, *A*_*inc*_ = *A*_*cold*_ − *A*_*hot*_ (purple hatched area in Fig. 2(a)) is the fraction of decay modified by electron-phonon scattering and *A*_*coh*_ = *A*_*cold*_ is the fraction not affected by this. This rate can be decomposed in a temperature-independent and a temperature-dependent contribution. The former is caused by lattice distortions due to the core excited state. The latter is proportional to the phonon population and thus, to the Bose-Einstein distribution^24^. Therefore, the evolution of the electron-phonon transfer rate with temperature can be written as:

2 $$R(T)={C}_{indep}+\frac{1}{{e}^{\frac{\langle {E}_{ph}\rangle }{kT}}}\cdot {C}_{dep}$$

where *C*_*indep*_ and *C*_*dep*_ correspond to the temperature independent and the temperature dependent contribution, respectively, and are used as fitting parameters. 〈*E*_*ph*_〉 is the average phonon energy.

From the peak areas, we deduce the electron-phonon spin-flip scattering rate vs. temperature and show it in Fig. 4. The error bars are determined by analyzing the fluctuation in the intensity when iterating data acquisition in similar conditions^16^. For nickel, the fit of our experimental data using Eq. (2), where 〈E_*ph*_〉 = 24 meV^25^, shows an almost linear increase of the momentum transfer rate from close to zero up to 0.15 fs^−1^ within our 300 K – 1200 K temperature range. For copper, where 〈*E*_*ph*_〉 = 20 meV^25^, no detectable spectral evolution with temperature is seen. As shown in Fig. 4(c), our method leads to an angular momentum transfer time scale at room temperature in the order of 50 fs for nickel. Even though this quantity, which refers to a process at the atomic scale, cannot be directly compared to the macroscopic demagnetization time measured using pump-probe experiments, it unambiguously demonstrate the importance of the Elliott-Yafet contribution in the demagnetization mechanism in nickel.

## Conclusion

To conclude, we present here a unique approach to measure the Elliott-Yafet contribution in the demagnetization process in nickel. It is based on static measurements and can therefore be applied in all synchrotron based facilities. It is also general to a broad range of magnetic materials^9,26^. Finally, our method can easily be applied for a better understanding of electron-phonon interactions in systems like (high-*T*_*C*_) superconductors^27,28^, graphene^29^, topological insulators^30,31^ or Weyl semimetals^32^.

## Methods

XES experiments were performed with the SolidFlexRIXS endstation on the high flux U49-2 PGM-1 beamline at BESSY II in the multibunch operating mode. Temperature dependent measurements were performed from room temperature up to almost the melting point of nickel and copper, reached by electron bombardment from a Tungsten filament. The base pressure was in the low 10^−8^ mbar range but rose up to the low 10^−6^ mbar range for the highest temperatures. Spectra were acquired with a GRAZE IV – type spectrometer equipped with a single photon counting microchannel plate (MCP) detector from Scienta. Samples, purchased at Matek, were placed on a tungsten sample plate. The temperature was measured using both a thermocouple on the sample plate and a pyrometer.

Density Functional Theory (DFT) calculations of the temperature effects on the X-ray emission spectra of nickel and copper were done using the linearized augmented plane-wave elk code (elk.sourceforge.net). The effect of thermal expansion on the emission spectrum was simulated by expanding the room temperature lattice parameters of Ni (3.52 Å) and Cu (3.58 Å) by 1.8 %. The product of the smallest muffin-tin radius and the largest G vector of the plane wave basis *R*_*MT*,*min*_ × *G*_*k*,*max*_ was set to 7. The ground state and spectrum calculations were performed on 40 × 40 × 40 k-point grids. The emission spectra were calculated in the random phase approximation. Effects of the initial (final) state core (valence) hole were neglected. Fermi surface smearing effects were accounted for by using the physical temperatures of 300 K and 1200 K in the calculation of the spectra. The calculations were performed with and without contributions from the bands crossing the Fermi level. The latter case simulates the effect of a spin-flip near the Fermi surface on the emission intensity.

## Supplementary information

Supplementary information

## Data Availability

All data are available upon reasonable request.
