# Supplementary material for: Measuring the atomic spin-flip scattering rate by x-ray emission spectroscopy
Source: Sci Rep. 2019 Jun 20;9:8977. doi: 10.1038/s41598-019-45242-8 (PMC6586882; doi:10.1038/s41598-019-45242-8)
Supplement: Supplementary file 1 — Supplementary information [file 41598_2019_45242_MOESM1_ESM.pdf]

Measuring the atomic spin-flip scattering rate by x-ray emission spectroscopy

Régis Decker[1,*,+], Artur Born[1,+], Robby Büchner[1,2], Kari Ruotsalainen[1], Christian Stråhlman[1], Stefan Neppl[1], Robert Haverkamp[1], Annette Pietzsch[1], and Alexander Föhlisch[1,2,]**

[1]Institute for Methods and Instrumentation for Synchrotron Radiation Research FG-ISRR, Helmholtz-Zentrum Berlin für Materialien und Energie Albert-Einstein-Strasse 15, 12489 Berlin, Germany
[2]Institut für Physik und Astronomie, Universität Potsdam, Karl-Liebknecht-Strasse 24-25, 14476 Potsdam, Germany

The supplementary material includes a summary of reported work on the demagnetization time scales in nickel, cobalt and iron, further details on the background subtraction and normalization method for the x-ray emission spectroscopy (XES) spectra presented in Figures 2 and 4 of the main text as well as the calculated effect of the temperature-driven lattice expansion and Fermi-Dirac smearing on the decay peak.

Since the pioneering work of Beaurepaire *et al.*, extensive work has been dedicated to the experimental determination of the demagnetization time scale in ferromagnets. In FIG. S1, we summarize the measured time scales of the 3d-ferromagnets nickel, cobalt and iron at room temperature and for various sample geometry, from several atomic layer thin films to bulk. All data originate from pump-probe experimental schemes, were the electron system is excited by a short laser pulse and subsequently measured by Magneto-Optical Kerr Effect (MOKE), X-ray Magnetic Circular Dichroism (XMCD), Photo-Electron Spectroscopy (PES) and Second-Harmonic Generation (SHG).

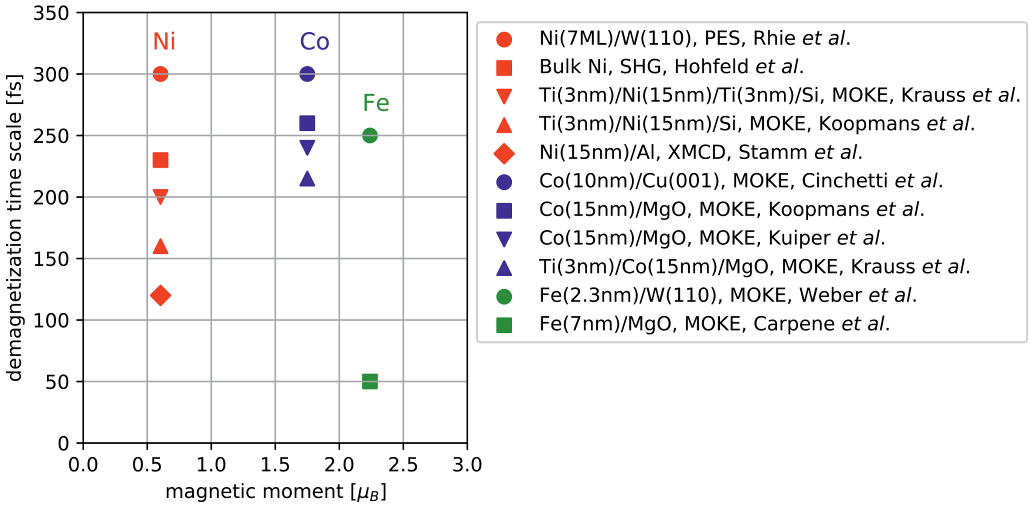

Figure S1. Reported demagnetization timescales vs. the magnetic moment, for Ni (red symbols)[1–5], Co (blue symbols)[1,2,6,7] and Fe (green symbols)[8,9] measured with pump-probe techniques and at room temperature.

FIG. S1 evidences the large discrepancy in the measured demagnetization times and shows the difficulty to establish clear time scales and to understand the demagnetization mechanisms. No clear dependence on the average magnetic moment of the element is observed. In addition, the sample geometry seems to have no influence. And even though different experimental techniques access different sample depths, no trend related to the technique employed is observed. A possible origin of this discrepancy is the different laser fluences, a higher fluence tends to result in a higher demagnetization time scale. This has been more recently described in terms of a higher demagnetization amplitude at higher fluences, using high-harmonic generation experiments[10].

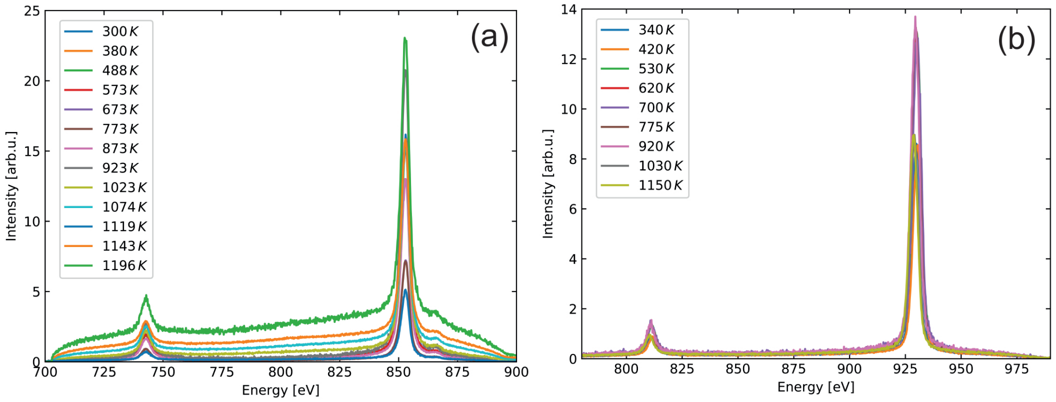

Figure S2. Raw XES spectra before background subtraction and normalization. (a) Raw nickel XES spectra for the temperature range 300 K – 1200 K. (b) Raw copper XES spectra for the temperature range 300 K – 1200 K.

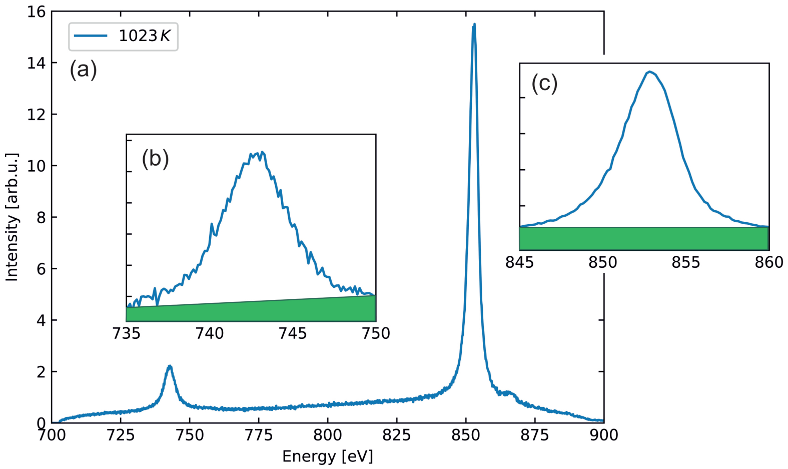

Figure S3. XES spectra normalization. (a) Raw XES spectrum for nickel at T = 1023 K. (b Detailed view of the $3s \to 2p_{3/2}$ lower-energy peak. (c) Detailed view of the $3d \to 2p_{3/2}$ higher-energy peak. The green areas in (b) and (c) are the areas removed in the normalization method.

FIG. S2 shows the raw XES spectra of (a) nickel and (b) copper for different temperatures and for an incident energy of 865 eV (nickel) and 940 eV (copper). We want here to give more details on the background subtraction and the spectra normalization method. The background is coming from the glowing filament and the radiations of the warm parts (sample holder, manipulator and even the chamber walls and detector entry) due to the several hours long heating of the samples needed to acquire the spectra. By measuring the background, *i.e.* by recording spectra without beam but with heating on in order to subtract it from the spectra, it appeared that it strongly depends on the settings of the heater and on the heating time. We concluded that this method of background subtraction was not reliable. Instead, we performed a linear background subtraction under the peak, with defined energy intervals. FIG. S3 illustrates this background subtraction method in the case of nickel. It consists of a linear fit of the background under the $3s \to 2p_{3/2}$ peak (735eV-750eV) and the $3d \to 2p_{3/2}$ peak (845eV-860eV). The background, *i.e.* the area below the linear fit (green area in the Figure S3) is removed for the proper analysis of data. We normalized afterwards each spectrum on its background–subtracted $3s \to 2p_{3/2}$ peak area. As discussed in the main text, this peak is considered as constant, due to the lack of free states in the $3s$ band. This leads to the spectra shown in FIG. 2, which shows the normalized spectra, including the background, which is still slightly visible, especially for nickel (see FIG. 2(a)). Since the background is still slightly present after normalization, the data points in FIG. 4 were derived from the area of the normalized $3d \to 2p_{3/2}$ peaks with the background subtracted. The error bars in FIG. 4 were estimated on the deviation of the $3d \to 2p_{3/2}$ peak area. The full spectrum is indeed the sum of 6 spectra acquired successively and the error is the deviation in the peak intensity in these 6 spectra. Using the mean deviation value and the error propagation law, we can estimate the error of the momentum transfer rate and the lifetime.

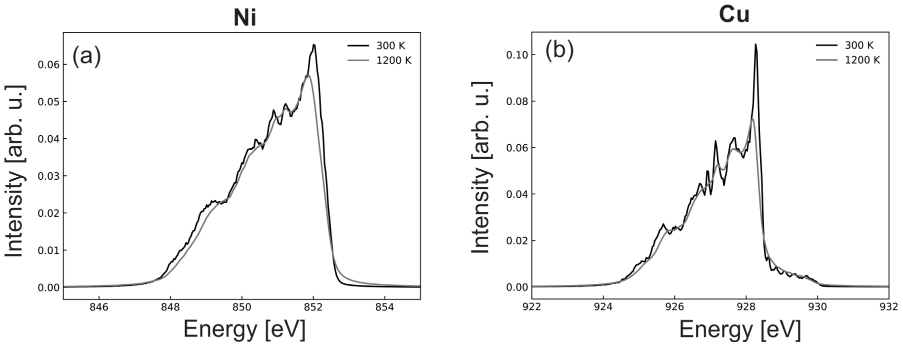

Figure S4. Calculated temperature dependence of the $3d \to 2p_{3/2}$ peak for nickel (a) and copper (b), considering the lattice expansion and the Fermi-Dirac smearing.

The question of the temperature effect on the XES spectra without scattering effects must be addressed. Indeed, an increase of the temperature leads to the lattice thermal expansion and to the Fermi-Dirac smearing. We show in FIG. S4 the calculated $3d \rightarrow 2p_{3/2}$ peak profile of nickel and copper for 300 K and 1200 K. In both nickel and copper, a reduction of the calculated peak area in the order of 5 % is found. The fact that this value is smaller than our uncertainty and that it is found for both nickel and copper further suggests than the origin of the experimentally observed emission peak reduction is the electronic scattering.

References

1. Krauß, M. *et al.* Ultrafast demagnetization of ferromagnetic transition metals: The role of the coulomb interaction. *Phys. Rev. B* **80**, 180407 (2009).
2. Koopmans, B. *et al.* Explaining the paradoxical diversity of ultrafast laser-induced demagnetization. *Nat. Mater.* **9**, 259 (2009).
3. Stamm, C. *et al.* Femtosecond modification of electron localization and transfer of angular momentum in nickel. *Nat. Mater.* **6**, 740 (2007).
4. Hohlfeld, J., Matthias, E., Knorren, R. & Bennemann, K. H. Nonequilibrium magnetization dynamics of nickel. *Phys. Rev. Lett.* **78**, 4861–4864 (1997).
5. Rhie, H.-S., Dürr, H. A. & Eberhardt, W. Femtosecond electron and spin dynamics in $w(110)$ films. *Phys. Rev. Lett.* **90**, 247201 (2003).
6. Kuiper, K. C. *et al.* Spin-orbit enhanced demagnetization rate in co/pt-multilayers. *Appl. Phys. Lett.* **105**, 202402 (2014).
7. Cinchetti, M. *et al.* Spin-flip processes and ultrafast magnetization dynamics in co: Unifying the microscopic and macroscopic view of femtosecond magnetism. *Phys. Rev. Lett.* **97**, 177201 (2006).
8. Weber, A. *et al.* Ultrafast demagnetization dynamics of thin fe/w(110) films: Comparison of time- and spin-resolved photoemission with time-resolved magneto-optic experiments. *Phys. Rev. B* **84**, 132412 (2011).
9. Carpene, E. *et al.* Dynamics of electron-magnon interaction and ultrafast demagnetization in thin iron films. *Phys. Rev. B* **78**, 174422 (2008).
10. La-O-Vorakiat, C. *et al.* Ultrafast demagnetization measurements using extreme ultraviolet light: Comparison of electronic and magnetic contributions. *Phys. Rev. X* **2**, 011005 (2012).
